# Real-world data in retinal diseases treated with anti-vascular endothelial growth factor (anti-VEGF) therapy – a systematic approach to identify and characterize data sources

**DOI:** 10.1186/s12886-019-1208-9

**Published:** 2019-10-16

**Authors:** Vincent Daien, Bora M. Eldem, James S. Talks, Jean-Francois Korobelnik, Paul Mitchell, Robert P. Finger, Taiji Sakamoto, Tien Yin Wong, Obaro Evuarherhe, Gemma Carter, Joao Carrasco

**Affiliations:** 1 Department of Ophthalmology, Gui De Chauliac Hospital, Montpellier, France; 2 Inserm, U1061, Montpellier, France; 3 The Save Sight Institute, Sydney Medical School, The University of Sydney, Sydney, NSW Australia; 4 Faculty of Medicine, Ophthalmology Department, Hacettepe University, Ankara, Turkey; 5 Department of Ophthalmology, Royal Victoria Infirmary, Newcastle upon Tyne, UK; 6 Service d’ophtalmologie, CHU Bordeaux, France - Univ. Bordeaux, Inserm, Bordeaux, France; 7 Bordeaux Population Health Research Center, team LEHA, Bordeaux, France; 8 Centre for Vision Research, Westmead Institute for Medical Research, University of Sydney, Sydney, NSW Australia; 9 Department of Ophthalmology, University of Bonn, Bonn, Germany; 10 Department of Ophthalmology, Kagoshima University Graduate School of Medical and Dental Sciences, Kagoshima, Japan; 11 Singapore Eye Research Institute, Singapore National Eye Centre, Singapore, Singapore; 12 Duke-NUS Medical School, Singapore, Singapore; 13 Oxford PharmaGenesis, Oxford, UK; 14 Bayer Consumer Care AG, Peter Merian-Strasse 84, 4052 Basel, Switzerland

**Keywords:** Retinal diseases, Age-related macular degeneration, Choroidal neovascularization, Diabetic macular edema, Retinal vein occlusion, Anti-VEGF, Real-world data

## Abstract

**Background:**

Real-world data (RWD) has been a valuable addition to the scientific literature regarding treatment pathways, clinical outcomes and characteristics of patients with retinal diseases in recent years. Registries, observational studies and patient databases are often used for real-world research. However, there is limited information for each data source on the design, consistency, data captured, limitations and usability for assessing research questions. Using a systematic approach, we identified RWD sources for patients with retinal diseases and assessed them for completeness of data relating to different outcomes.

**Methods:**

A systematic literature review was carried out to identify RWD sources for patients with retinal disease. Potentially relevant articles published between 2006 and 2016 were screened following electronic searches in Embase and MEDLINE. Congress and supplementary searches were undertaken to identify RWD sources that may not be referenced in full publications. For each data source, availability and quantity of data on baseline status, clinical outcomes, treatment and management, safety, and patient-reported and economic burden were assessed using a bespoke completeness assessment tool based on International Consortium for Health Outcomes Measurement guidelines for macular degeneration. Completeness of data for each area of interest in each data source was assessed and rated using a ‘good–moderate–poor’ rating system based on availability and quantity of available data. Each data source was then given an overall score based on its score for each of the 7 areas of interest.

**Results:**

A total of 128 RWD sources from 32 countries were identified. Of the identified sources, 64 sources from 16 countries of interest were analyzed. Most of these sources provided information on baseline status and clinical outcomes and treatment, but few collected data on economic and patient-reported burden. Of the RWD sources analyzed, 10 scored highly in the overall completeness assessment, collecting data on most or all of the areas of interest; these sources are considered to be robust data sources for performing ophthalmology real-world studies.

**Conclusions:**

The study provides a comprehensive list of RWD sources for patients with retinal disease, many of which will be useful for conducting real-world studies in the field of ophthalmology.

**Electronic supplementary material:**

The online version of this article (10.1186/s12886-019-1208-9) contains supplementary material, which is available to authorized users.

## Background

### Retinal diseases

Retinal diseases, which are often characterized by leakage of fluid, hemorrhage and fibrous scarring in the eye, include wet age-related macular degeneration (wAMD), diabetic macular edema (DME), retinal vein occlusion (RVO) and choroidal neovascularization (CNV) not secondary to AMD. These diseases are major causes of visual impairment and blindness worldwide [1, 2]. Retinal diseases can cause irreversible loss of visual acuity, which can have a major impact on patients’ vision-related quality of life and overall wellbeing, often leading to anxiety and depression, an increase in healthcare resource utilization and high patient support costs [3–9]. Patients commonly experience an increase in feelings of social isolation and discontinue employment, which can, in turn, lead to a loss of income and independence [10].

### Treatment options

Over the past decade, anti-vascular endothelial growth factor (anti-VEGF) therapy has replaced photodynamic therapy and laser photocoagulation as the standard of care for treatment of these diseases [11]. Anti-VEGF antibodies prevent the growth of new blood vessels through inhibition of the physiological effects of VEGF. The main anti-VEGF therapies in current use are bevacizumab (Avastin), ranibizumab (Lucentis) and aflibercept (Eylea). Avastin is a full-length, recombinant, humanized, anti-VEGF monoclonal antibody, which was initially designed and approved for the treatment of various solid tumors; it is now used off-label to treat retinal disease [12–14]. Lucentis is a humanized, recombinant antibody fragment of a monoclonal antibody active against all VEGF A isoforms; it has been approved for use in adults with wAMD, CNV, DME and RVO [15]. Eylea is a recombinant fusion protein comprising the key VEGF-binding domains of human VEGF receptors 1 and 2 fused to the constant region of human immunoglobulin G1. Eylea acts as a soluble decoy receptor that binds VEGF-A and placenta growth factor with higher affinity than naturally occurring VEGF receptors and is approved for the treatment of wAMD, CNV, DME and RVO [16].

### Real-world data for retinal diseases

Real-world data (RWD) comprises findings from various observational study types and can provide vital information about the effectiveness of a treatment in clinical practice. RWD sources consist of diverse cohorts of patients, including those that are normally excluded from randomized controlled trials (RCTs), and can therefore provide insight into the clinical effectiveness of a treatment in various subgroups of patients in a real-world setting [17, 18]. Evidence from RWD sources has provided a greater understanding of the long-term safety, effectiveness in clinical practice and utilization patterns of anti-VEGF therapy, in addition to providing important information on the patients as well as the disease [19–21].

The use of RWD to understand retinal diseases and current treatment options is growing. Since the approval of the anti-VEGF therapies, many countries and regions have set up retinal registries, observational studies and databases containing information on patients with retinal diseases. However, the design and data captured within these multiple data sources are not consistent, and their usability and limitations in assessing important research questions may not be fully understood. A valuable addition to the scientific literature, therefore, would be a systematic study in which all available RWD sources for retinal diseases were identified and collated, and which also included a qualitative assessment of the data collected from each source. In addition, an evaluation of the strengths and limitations of each database would be useful to identify the most suitable RWD sources to perform future real-world studies, and to appraise the completeness of existing real-world studies. The objective of this study was to conduct a systematic literature review (SLR) to identify and compile information about existing RWD sources that include patients with retinal diseases treated with anti-VEGF therapies, and to assess systematically the completeness of the data available in these sources.

## Methods

The SLR was conducted in 2 stages: in the first stage, data sources (including multinational and international sources) outside of the USA were identified by applying a restriction to the search terms; and, in the second stage, a follow-up search was carried out specifically to identify articles describing retinal disease-related data sources within the USA.

Because this study did not involve the collection or use of individually identifiable data, Institutional Review Board review or approval was not required and therefore no formal review protocol exists for this study.

### Citation screening and full text review

Identified articles were screened manually based on the title and abstract in accordance with 2009 Preferred Reporting Items for Systematic Reviews and Meta-Analyses (PRISMA) guidelines [22].

Articles were eligible if they described an active RWD source for retinal diseases (wAMD, DME, RVO and myopic CNV) and were published from 2006 onwards. Eligible publications for all countries were tagged, but only those for the multinational data sources and countries of interest were reviewed in further detail. The countries of interest were Australia, Brazil, Canada, China, Denmark, Finland, France, Germany, Iceland, Italy, Japan, Norway, Spain, Sweden, the UK and the USA. Articles describing data sources that focused on patients under 18 years of age or patients with other retinal diseases were excluded. Case studies, RCTs, pooled analyses, economic evaluations and non-English articles were also excluded. Full-text versions of all articles meeting the eligibility criteria were reviewed to confirm whether the patients in the reported study were recruited from an established data source. Some data sources were described in more than one publication.

### Data extraction

Articles describing RWD sources were further analyzed to extract data on the availability of specific variables and outcomes of interest (Table 1). General information on each data source was extracted, such as country, data type, patient types and numbers, and outcomes captured. Other details relating to the ownership, accessibility and availability of the data source were also recorded (Additional file 1: Table S1).

**Table 1:** Completeness of data recorded in reported data sources

Country	Data source name (type)	Baseline status	Outcomes					
			Clinical	Treatment	Anti-VEGF	Safety	Human	Economic
Australia	Fight Retinal Blindness (active disease registry)	+++	++	+++	+++	+++	–	–
	Australian Heart Eye Study (prospective cohort study)	++	–	+	+	–	–	–
	Blue Mountains Eye Study (prospective cohort study)	+++		+	+	–	–	–
	Macular Disease Foundation (active disease registry)	–	–	+	+++	–	++	–
	Melbourne Collaborative Cohort Study (prospective cohort study)	+++	–	+	+	–	–	–
Canada	Southwestern Ontario Database (passive disease registry)	+++	+++	+++	+++	+++	+++	+++
	British Columbia Ministry of Health Databases (administrative/claims database)	++	–	++	+++	–	–	–
	Quebec prescription and medical claims databases (administrative/claims database)	++	–	+	–	+++	–	++
China	Beijing Eye Study (population-based longitudinal study)	+++	+++	+++	–	–	–	–
Denmark	Four referral centres in Denmark (unnamed – electronic medical records)	++	–	+	–	–	+++	–
France	Creteil Intercommunal University Hospital Eye Clinic (prospective cohort study)	++	+++	++	+++	–	++	+++
	Retina France (cross-sectional study)	+++	–	++	+++	–	+++	+++
	ALIENOR (prospective cohort study)	+++	+	+++	+	–	+++	–
Germany	OCEAN (prospective cohort study)	++	+++	+++	+++	–	+++	++
	CAPTAIN (retrospective cohort study)	+++	–	+++	+++	–	++	+++
	Bonn Ophthalmology online network (active disease registry)	+	++	++	+++	–	–	+++
	Gutenberg Health Study (prospective cohort study)	+++	+++	+	+	–	–	–
	FAM study (prospective cohort study)	+++	–	++	+	–	–	–
	Landschaftsverband Rheinland database (active disease registry)	++	–	–	+	–	–	–
Iceland	Age, Gene/Environment Susceptibility–Reykjavik Study (prospective cohort study)	+++	–	–	+	–	–	–
Japan	Japan Medical Data Centre (administrative/claims database)	++	–	++	+++	–	–	+++
	Hatoyama Cohort Study (prospective cohort study)	+++	–	+	–	–	++	++
	Hisayama study (prospective cohort study)	++	–	+	+	–	–	–
Multinational	AURA study	+++	+++	+++	+++	+++	–	+++
	The EPICOHORT study	++	+++	+++	+++	+++	–	–
	European Genetics Database	+++	+++	+++	+++	–	–	++
	IRISS	+	++	+	–	+++	–	–
	LUMINOUS study	++	+++	+++	+++	+++	–	+++
	VigiBase	+++	++	++	+++	+++	–	–
Norway	Tromso Eye Study (population-based longitudinal study)	+++	–	+	+	–	–	–
Sweden	Swedish Macula Register (active disease registry)	+++	+++	+++	+++	+++	+++	+++
	Swedish Lucentis Quality Registry (active disease registry)	++	+++	+++	+++	+++	+++	++
UK	British Ophthalmological Surveillance Unit (electronic medical records)	++	++	+++	+++	+++	–	+++
	Medisoft (electronic medical records)	+++	+++	+++	+++	+++	+++	+++
	Scotland intravitreal ranibizumab treatment register (unnamed – electronic medical records)	+++	+++	+++	+++	+++	–	+++
	UK Age-Related Macular Degeneration Electronic Medical Record Users Group (retrospective cohort study)	++	+++	+++	+++	+++	–	+++
	Unnamed tertiary referral clinic (active disease registry)	+++	+++	+++	+++	–	–	+++
	Gloucestershire NHS ophthalmology department (electronic medical records)	++	+++	+++	+++	–	–	–
	Medical Retina Clinic at King’s College London (electronic medical records)	+++	+++	+++	+++	–	–	++
	Medical Retina Service, St Thomas’ Hospital (electronic medical records)	++	+++	++	+++	–	–	+++
	Belfast Health and Social Care Trust database (unnamed - electronic medical records)	++	–	++	+++	+++	–	–
	The National Ophthalmology Database (active disease registry)	++	–	–	+	–	–	–
	Grampian University Hospitals (electronic medical records)	++	–	–	+	–	–	–
	English National Hospital Episode Statistics (electronic medical records)	++	–	–	–	–	–	–
USA	Duke University Eye Center (active disease registry)	+++	+++	+++	+++	+++	+++	++
	Bascom Palmer Eye Institute (active disease registry)	++	+++	+++	+++	+++	–	–
	IMS Health (administrative/claims database)	++	–	+++	+++	+++	–	+++
	Wilmer Eye Institute (electronic medical records)	++	+++	+++	+++	+++	–	–
	Doheny Eye Institute (active disease registry)	+++	+++	+++	+++	–	–	–
	VRMNY (electronic medical records)	+++	+++	+++	+++	–	–	–
	Wills Eye Hospital (electronic medical records)	+++	+++	+++	+++	–	–	–
	A2ASDOCT (prospective observational study)	+++	+++	+++	–	–	–	–
	Shiley Eye Center (prospective observational study)	++	+++	+++	+++	–	++	++
	BRFSS (questionnaire/survey)	+++	–	+	–	–	++	++
	i3 InVision Data Mart (electronic medical records)	+++	–	++	+++	–	–	–
	New England Eye Center (retrospective observational study)	++	+++	+++	–	–	–	–
	SOF/IAMD (prospective observational study)	+++	+++	+	+	–	–	–
	Beaver Dam Eye Study (prospective observational study)	+++	+++	+	–	–	–	–
	LALES (prospective observational study)	+++	–	+	–	–	+++	–
	Nurses’ Health Study (prospective observational study)	+++	–	++	+	–	–	–
	CAREDS (prospective observational study)	+++	–	+	–	–	–	–
	MESA	+++	–	+	–	–	–	–
	NHANES	+++	–	+	–	–	–	–

+++, ‘good’; ++. ‘moderate’; +, ‘poor’; −, ‘unknown’. A2ASDOCT, AREDS (Age-Related Eye Disease Study) 2 Ancillary Spectral Domain Optical Coherence Tomography Study; ALIENOR, Antioxydants, LIpides Essentiels, Nutrition et maladies OculaiRes; AURA, Study to Assess the Effectiveness of Existing Anti vascular Endothelial Growth Factor (Anti VEGF) in Patients With Wet Age-related Macular Degeneration; BRFSS, Behavioral Risk Factor Surveillance System; CAPTAIN, the comparison of applied ophthalmological tests for suspected age-related macular disease patients; CAREDS, Carotenoids in Age-Related Eye Disease Study; EPICOHORT, study to assess the safety profile, treatment patterns, and efficacy of ranibizumab in real-life clinical setting/routine clinical practice; FAM, Fundus Autofluorescence in Age-related Macular Degeneration Study; IMS Health, IMS Health Real-World Data Medical Claims database; IRISS, The ILUVIEN Registry Safety Study; LALES, Los Angeles Latino Eye Study; LUMINOUS, study to evaluate the long-term safety, efficacy, treatment patterns and health-related quality of life outcomes in patients treated with ranibizumab in routine practice; MESA, Multiethnic Study of Atherosclerosis; NHANES, US National Health and Nutrition Examination Survey; NHS, UK National Health Service; OCEAN, Observation of Treatment Patterns With Lucentis in Approved Indications; SOF/IAMD, Study of Osteoporotic Fractures/Incidence of AMD study; UK, United Kingdom; UK-AMD-EMR, UK Age-Related Macular Degeneration Electronic Medical Record Users Group; USA, United States of America; VEGF, vascular endothelial growth factor; VRMNY, Vitreous Retina Macula Consultants of New York

### Assessing the availability and completeness of the data collected

The variables and outcomes of interest included in the qualitative assessment were defined based on the recommendations made by the International Consortium for Health Outcomes Measurement (ICHOM) for Macular Degeneration (outlined by Rodrigues et al. 2016) [23]. The outcomes of interest were grouped into 7 categories: patient baseline status (patient characteristics and baseline disease information); clinical outcomes; patient treatment and management, including laboratory assessments; anti-VEGF therapy status (naïve or experienced); safety outcomes; patient-reported burden; and economic burden.

### Supplementary searches for each data source

For each data source, information from identified publications was supplemented by additional internet searches for supporting resources, such as websites and published factsheets. This approach was particularly useful for gathering data source contact details and general information.

### Direct contact with data sources

It is not always possible to assess fully the suitability of data sources because published studies may not identify them by name, and information available on the data source websites may be limited. Thus, we contacted the administrators of a shortlist of identified data sources for further information on the data collected and to assess the accessibility of the data to external researchers. Up to 3 attempts were made to establish contact with the data source personnel via email or telephone. If no response was received after the third attempt at contact, no further action was taken to collect additional information.

### Evaluating the completeness of the data collected

**Fig. 1:**
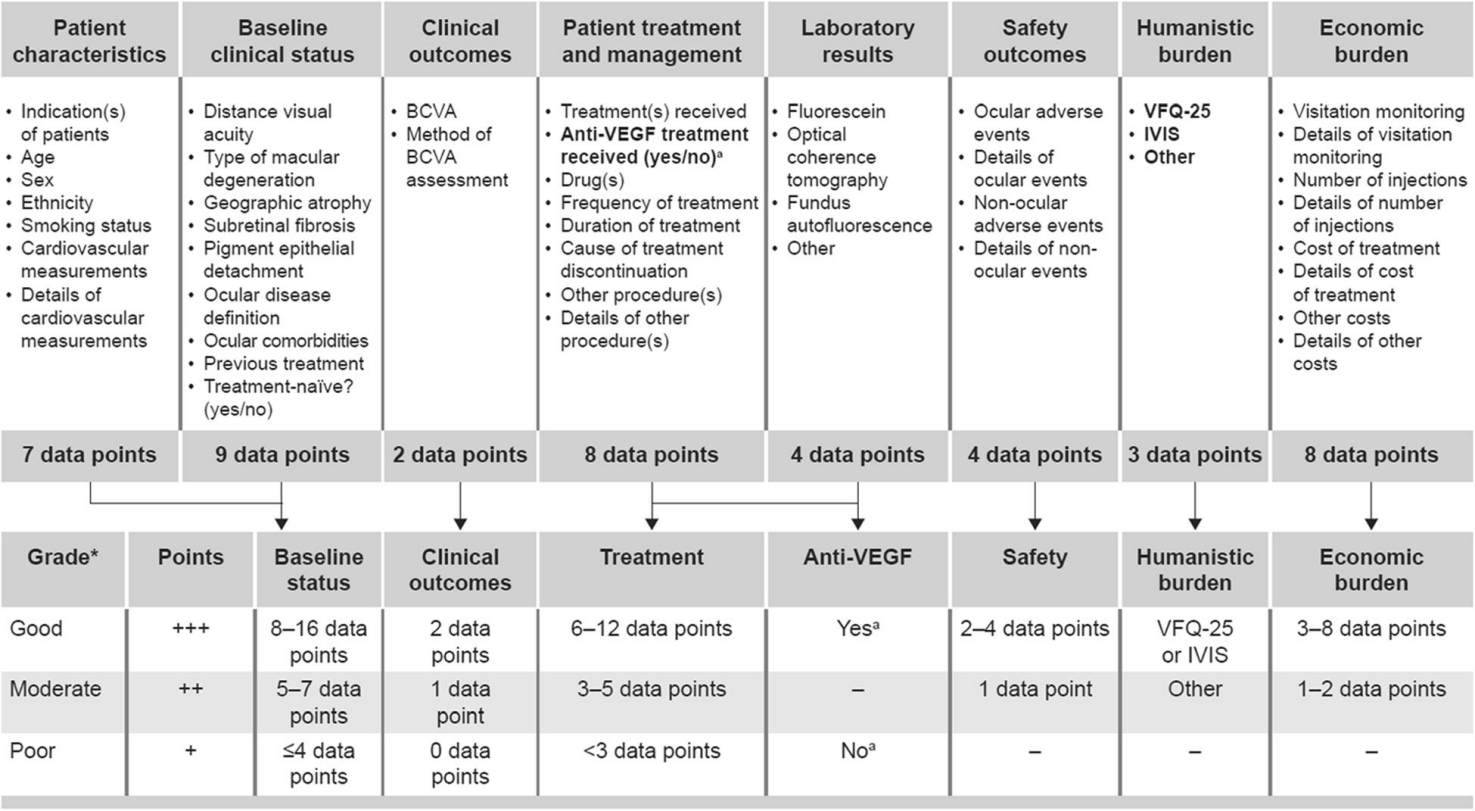
Data source completeness assessment criteria. *Outcomes data received through direct contact were classified as good if the data source administrator confirmed its availability. BCVA, best-corrected visual acuity; IVIS, Impact of Visual Impairment Scale; VEGF, vascular endothelial growth factor; VFQ-25, 25-item Visual Function Questionnaire

To appraise the information gathered for each data source, an assessment tool was developed that was tailored to the outcomes of interest based on the ICHOM guidelines. Using this tool, each topic of interest was rated using a ‘good–moderate–poor’ rating system based on the quantity and completeness of available information for each data source (Fig. 1). For example, there were 16 potential data points/variables related to the patient baseline status. Data sources that reported 8–16 data points were considered to be ‘good’ with regard to baseline status, sources with 5–7 data points were classified as ‘moderate’ and sources with less than 5 data points were classified as ‘poor’.

After the data sources were rated for each of the topics of interest, an overall point was assigned to each to reflect its general completeness (see Fig. 1). For each outcome of interest, a source rated ‘good’ received 3 points, a source rated ‘moderate’ received 2 points and a source rated ‘poor’ received 1 point. As there are a total of 7 outcomes of interest, each data source could score a maximum of 21 points.

## Results

### Search results

**Fig. 2:**
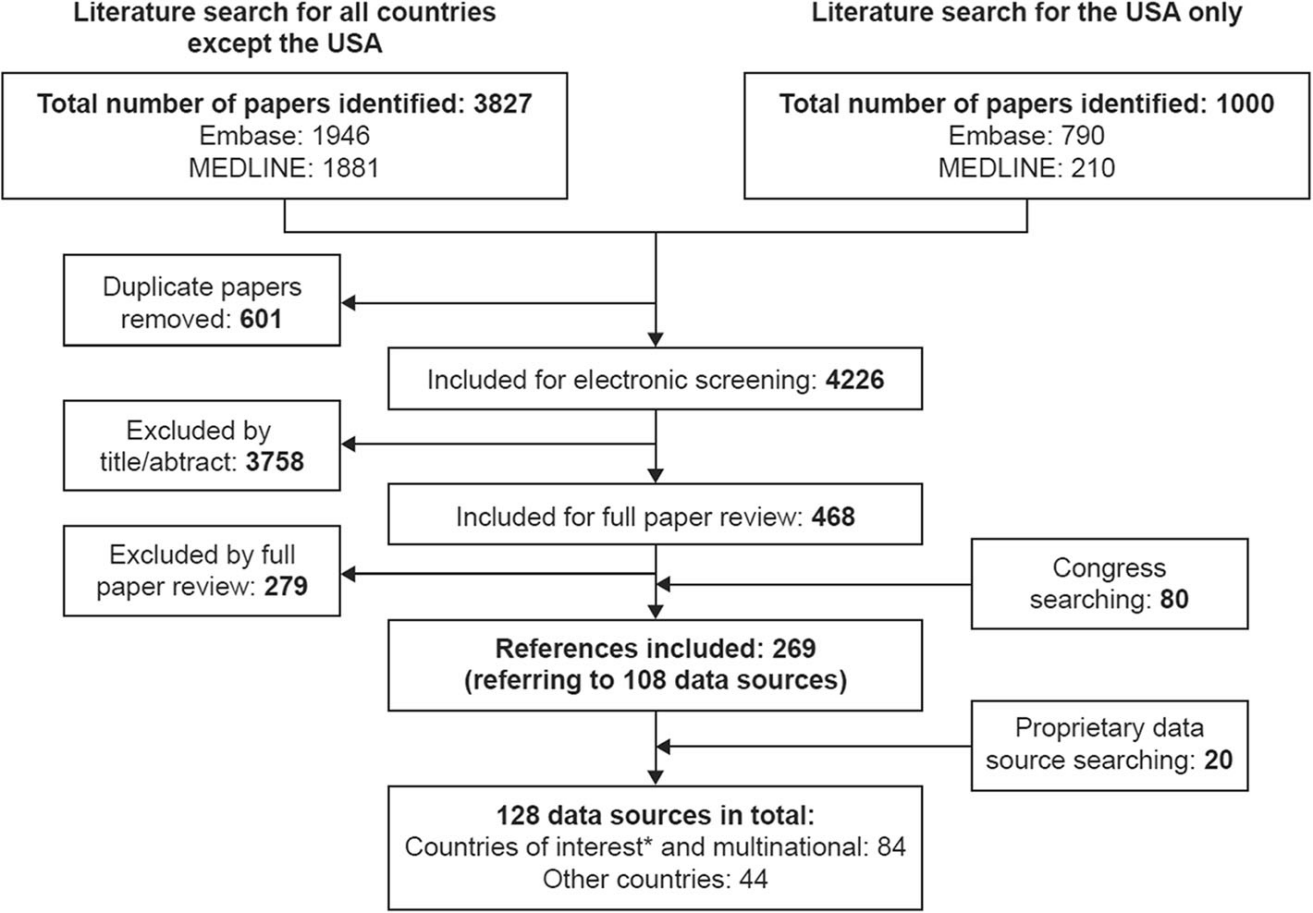
PRISMA diagram. *Countries of interest: Australia, Brazil, Canada, China, Denmark, Finland, France, Germany, Iceland, Italy, Japan, Norway, Spain, Sweden, the UK and the USA. PRISMA, Preferred Reporting Items for Systematic Reviews and Meta-Analyses

In the first stage of the SLR, the electronic searches identified 3827 articles that included information suggestive of a retinal disease data source. Screening of the full papers for data sources combined with findings from the supplementary searches resulted in the inclusion of a total of 119 articles referring to 89 data sources outside the USA (Fig. 2). In the second stage, the follow-up searches identified 1000 articles, of which 92 described 19 data sources in the USA (Fig. 2). In total, 108 RWD sources for retinal diseases were identified in our systematic search. Supplementary searches of proprietary databases identified 20 further data sources that were not described in publications.

**Fig. 3:**
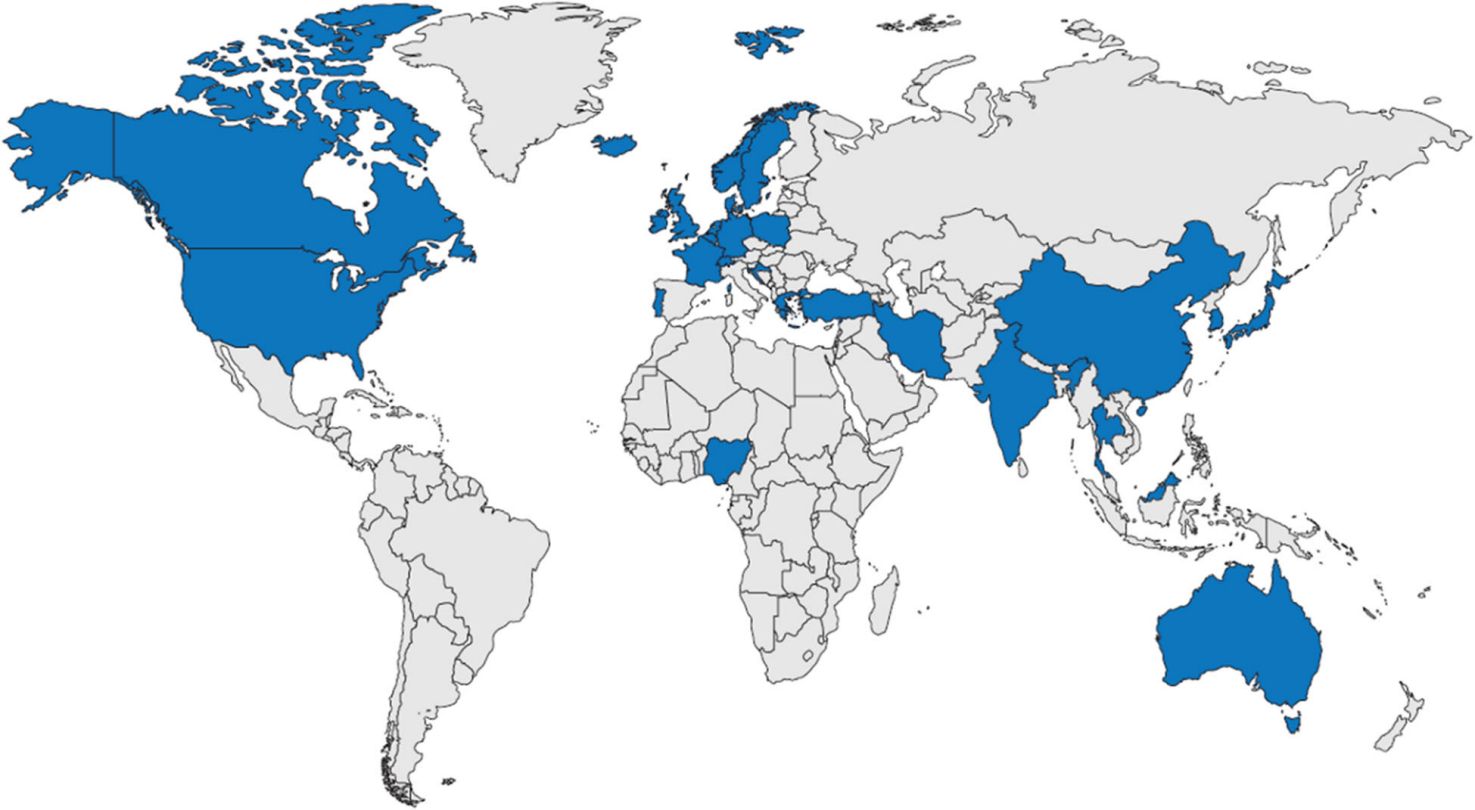
Geographical distribution of identified data sources^*^. Countries with identified data sources: Australia, Barbados, Belgium, Canada, China, Croatia, Czech Republic, Germany, Denmark, France, Greece, Iceland, India, Iran, Israel, Japan, Malaysia, The Netherlands, New Zealand, Nigeria, Norway, Poland, Portugal, Republic of Ireland, Singapore, South Korea, Sweden, Switzerland, Taiwan, Thailand, Turkey, the UK and the USA. ^*^ This is an original image created by the authors

In total, the analyses identified 128 RWD sources for retinal diseases. Of these, 6 data sources were multinational/international and the remaining 122 focused on a particular country. Overall, RWD sources from 32 countries were identified (Fig. 3). Information in the non-proprietary data sources from the countries of interest (*n* = 64) was subsequently extracted from the relevant studies (*n* = 212) and included in the assessment of completeness. Administrators for 42 of these data sources were contacted by email or telephone, 8 of which responded positively to a request for information: National Ophthalmology Database (UK); Australian Heart Eye Study; Southwestern Ontario Database (Canada); English National Hospital Episode Statistics (UK); Japan Medical Data Center; World Health Organization Database of Adverse Drug Reactions; Swedish Macula Register; and the Medical Retina Service in St Thomas’ Hospital (UK). The data already extracted for these sources were supplemented with the information received from direct contact.

### Data source characteristics

**Fig. 4:**
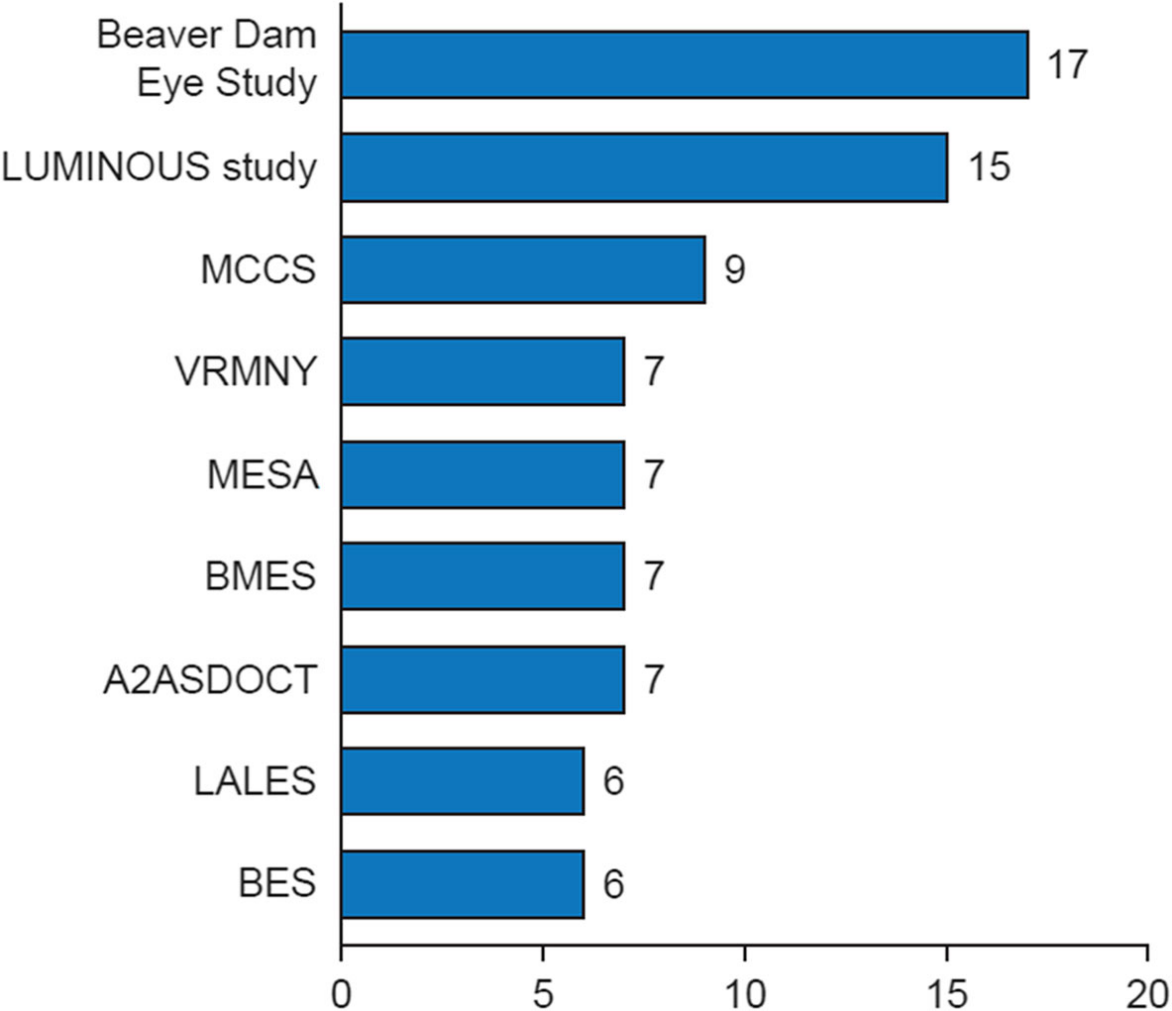
Number of publications per data source^*^. A2ASDOCT, AREDS (Age-Related Eye Disease Study) 2 Ancillary Spectral Domain Optical Coherence Tomography Study; BES, Beijing Eye Study; BMES, Blue Mountains Eye Study; LALES, Los Angeles Latino Eye Study; LUMINOUS, study to evaluate the long-term safety, efficacy, treatment patterns and health-related quality of life outcomes in patients treated with ranibizumab in routine practice; MCCS, Melbourne Collaborative Cohort Study; MESA, Multiethnic Study of Atherosclerosis; VRMNY, Vitreous Retina Macula Consultants of New York. ^*^ Data sources with more than 5 relevant publications

Among the 64 data sources included for extraction and assessment, 33 were referred to in 1 or 2 publications. The multinational LUMINOUS program and the Beaver Dam Eye Study in the USA were the most widely cited data sources, referred to in 15 and 17 publications, respectively (Fig. 4). The USA had the highest number of RWD sources with 19, followed by 13 for the UK and 6 for Germany. The most common types of RWD sources were prospective cohort studies (*n* = 21), electronic medical records (*n* = 14) and active disease registries (in which data are collected as ‘policy’, *n* = 14). The other identified data sources were administrative/claims databases (*n* = 4), retrospective cohort studies (*n* = 4), population-based longitudinal studies (*n* = 2), passive disease registries (in which data are collected upon recommendation or out of interest; *n* = 2), surveys (*n* = 2) and a cross-sectional study (*n* = 1). The identified data sources varied widely in terms of numbers of patients included. Two data sources included information for less than 100 patients and an additional 2 data sources included data for more than 100,000 patients. The Japan Medical Data Center had information on more than 700,000 patients with AMD, the largest number of relevant patients in a single database, followed by the Nurses’ Health Study (USA) with more than 280,000 participants. However, the IMS Health Real-World Data Medical Claims database (USA) contained the highest cumulative number of patients, with about 1 billion professional fee claims per year. The i3 InVision Data Mart (USA) included data from more than 15 million annual lives and Wills Eye Hospital (USA) included data from more than 250,000 patients each year.

### Outcomes reported and assessment of completeness

**Fig. 5:**
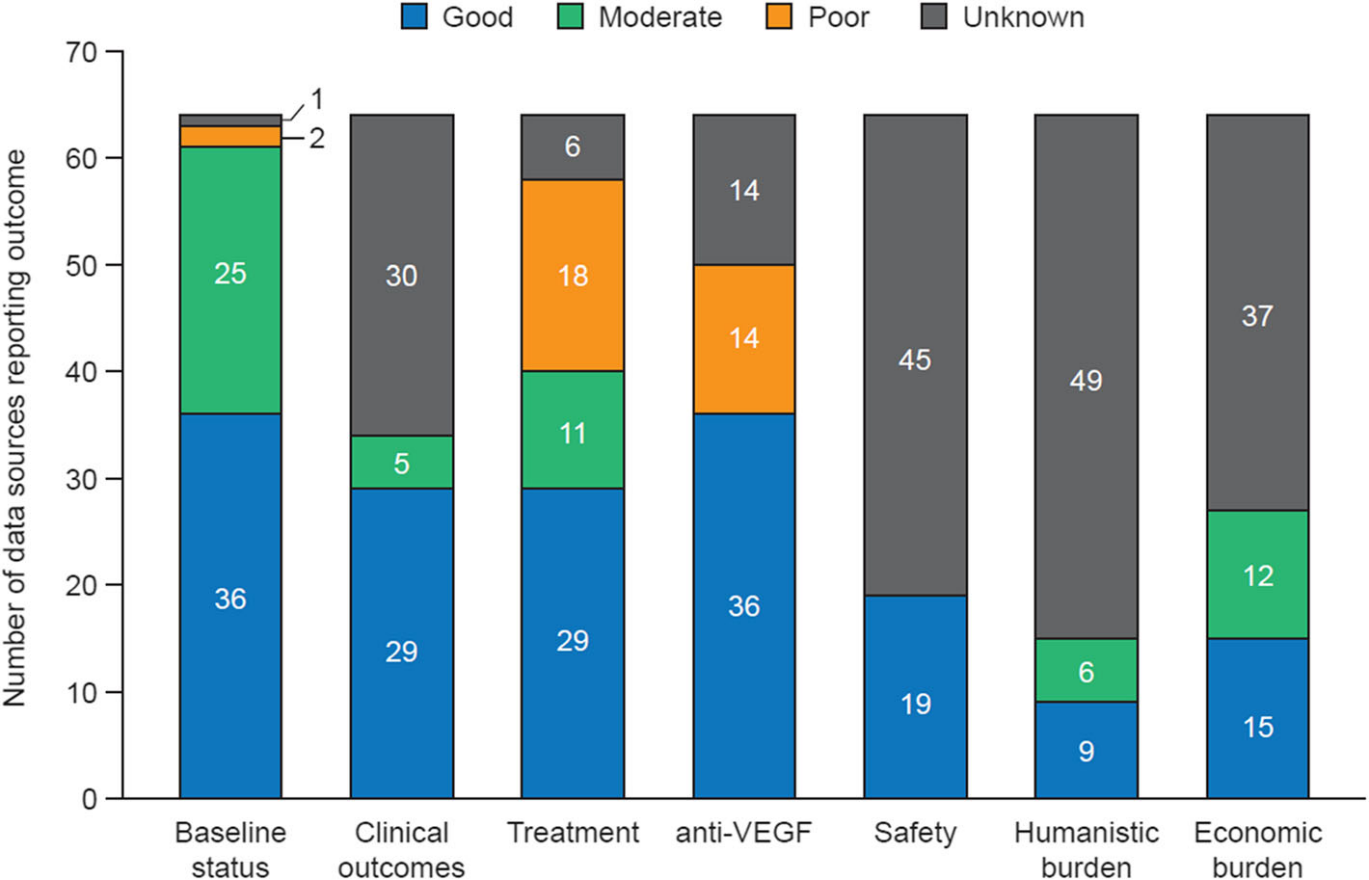
Completeness of identified data sources. VEGF, vascular endothelial growth factor

Overall, there was significant heterogeneity in outcomes collected between registries, with the majority of RWD sources providing information on patient baseline status, clinical outcomes and treatment outcomes (Fig. 5). All but 1 of the analyzed data sources recorded information on patient baseline status (patient characteristics and baseline disease information: 98% [63/64]); the majority included ‘good’ or ‘moderate’ data on baseline status (97% [61/63]). Most data sources collected information on patient age (60/64) and sex (61/64), but less than half collected information on ethnicity (28/64), smoking status (24/64) and cardiovascular measurements (28/64). Regarding baseline clinical status, distance visual acuity at baseline was recorded in two-thirds of identified data sources (68% [44/64]). Many of the data sources also recorded geographic atrophy (25/64), subretinal fibrosis (20/64), pigment epithelial detachment (24/64) and ocular comorbidities (25/64), and around a third recorded previous treatment information (34% [22/64]).

Around half of the analyzed data sources (56% [34/64]) recorded data on clinical outcomes (best-corrected visual acuity [BCVA]), most of which also reported the method of BCVA assessment (79% [27/34]). All of the data sources recorded BCVA using the Early Treatment of Diabetic Retinopathy Study protocol, Snellen chart or the logMAR chart (where visual acuity is scored with reference to the logarithm of the minimum angle of resolution). Data on patient treatment, including laboratory assessments, were available in most sources (90% [58/64]). For treatment data, 60% of data sources (39/64) recorded treatments received, most of which included information on anti-VEGF therapy (92% [36/39]). Further treatment data, such as frequency of treatment, duration of treatment and cause of treatment discontinuation, were recorded in 12, 11 and 5 of these data sources, respectively. Safety outcomes, including ocular and non-ocular adverse events, were reported in few data sources (30% [19/64]); most of these (16/19) recorded ocular adverse events, including endophthalmitis, hemorrhage and corneal abrasion.

When assessing the availability of patient-reported burden outcomes, quality-of-life data were available in a quarter of identified data sources (25% [16/64]). Of these, 7/16 assessed vision-related quality of life using the 25-item Visual Function Questionnaire. The remaining data sources used other instruments, including: the Geriatric Depression Scale short form, with 15 items; the basic Activities of Daily Living scale, with 5 items; and the social function questionnaire in the Hatoyama Cohort Study. Economic burden data were collected in 41% (27/64) of the identified data sources. More than half of these 27 sources recorded the number of injections (22/27) or number of visits (17/27).

Overall, 10 data sources scored well across the different variables, gaining at least 16 points out of a possible total of 21. These 10 sources are considered to be robust data sources for performing ophthalmology real-world studies looking at 5 or more of the 7 outcomes of interest. The top 10 data sources identified were: Southwestern Ontario Database (Canada; 21 points); Swedish Macula Register (21 points); Medisoft (UK; 21 points); Duke University Eye Centre (USA; 20 points); the Swedish Lucentis Quality Registry (19 points); the Study to Assess the Effectiveness of Existing Anti vascular Endothelial Growth Factor (anti VEGF) in Patients With Wet Age-related Macular Degeneration (AURA study; multinational; 18 points); an unnamed ranibizumab treatment register in south-east Scotland (UK; 18 points); the LUMINOUS program (multinational; 17 points); the UK Age-Related Macular Degeneration Electronic Medical Record System Users Group (17 points); and the Observation of Treatment Patterns With Lucentis in Approved Indications (OCEAN study; Germany; 16 points).

## Discussion

### Summary of the findings

To our knowledge, this is the first study to identify and evaluate systematically the completeness of retinal disease-related RWD sources. Our study identified many sources of RWD that provide insightful and comprehensive evidence on retinal diseases and their treatments. Of the 128 data sources identified, 64 were analyzed in detail, 10 of which were considered to be useful sources for carrying out real-world studies for most or all of the outcomes of interest, including patient information, clinical and safety outcomes, treatment information, and patient-reported and economic burden.

### Value of real-world evidence in ophthalmology

RWD provides information about the effectiveness of a treatment in a real-life setting in a wider patient population than in a RCT. RWD adds to the evidence base for a disease and its treatment by providing information on topics such as long-term safety, treatment patterns, disease progression and burden of illness, which are not designed to be assessed in clinical trials. RWD are increasingly being considered alongside data from RCTs in healthcare decision-making. For instance, the US Food and Drug Administration uses RWD in specific contexts (for example, with devices) based on existing evidentiary standards. Similarly, the National Institute for Health and Care Excellence (NICE) in the UK considers RWD in addition to more traditional clinical evidence in technology appraisals. Therefore, robust data sources that permit high-quality, observational research on outcomes of interest are important to meet the increasing demands for real-world evidence from healthcare decision-makers. Among the data sources analyzed in this study, we confirmed that the Southwestern Ontario Database (Canada), the Swedish Macula Register and Medisoft (UK) record data on all outcomes of interest as recommended by the ICHOM guidelines, each scoring the full 21 points. As such, these 3 RWD sources will be useful for developing comprehensive real-world studies.

### Characteristics of data sources identified

RWD have provided important insights in the ophthalmology field, particularly in terms of treatment outcomes for anti-VEGF regimens, by permitting the assessment of outcomes for patients who may not typically be included in RCTs. Our study identified that there are many sources of RWD for retinal diseases and treatments worldwide. A high proportion of the sources recorded data on patient treatment and management (90% [58/64]), with 60% of these sources capturing information on whether or not patients had received anti-VEGF therapy. In addition to treatment information, many of the data sources provided information on the patient and their disease characteristics, as well as clinical outcomes. In contrast, relatively few data sources collected safety outcomes, patient-reported burden and economic data. In total, 10 data sources (6 from Europe, 1 from Canada, 2 multinational and 1 from the USA) provide good or moderate data for multiple outcomes of interest. It is important for retinal data sources to measure multiple outcomes to be able to provide a true sense of treatment effectiveness in clinical practice; measuring visual acuity, for example, is meaningless without capturing information on treatment patterns. By providing comprehensive information on clinical, treatment, safety, human and economic outcomes, these data sources can be considered to be robust for performing real-world studies.

### Outcomes assessed

To assess the completeness of the identified data sources, data items for outcomes of interest were selected and sources were scored on the presence or completeness of the data captured. For some outcomes of interest, our study found that the type and depth of information available differed across the data sources. This might reflect differences in how the data are captured and/or reported in publications. To permit comparison across real-world sources, it is important to standardize the types of data items collected and the outcome definitions used. In addition, the most suitable and relevant data items should be captured. Data items collected for appraisal of the data sources in this study were based on recommendations by the ICHOM guidelines, as outlined by Rodrigues et al. (2016) [23]. The data items collected in the completeness assessment in our study is based on recommendations for outcomes that matter most to patients and which therefore can be considered as robust data items for assessment [23]. Although many RWD sources capture the data items recommended by the ICHOM guidelines, this study has highlighted that several sources omit key outcomes measures. Therefore, there is a need for new RWD sources that standardize and align the data collected.

### Strengths and limitations

This is the first analysis to identify and assess all worldwide RWD sources for patients with retinal disease treated with anti-VEGF therapy. This analysis used a robust and systematic approach to identify all the data sources available, and collected data items that were recommended in the ICHOM guidelines. The completeness assessment profile for each data source was based on previously published methodology for a similar study assessing the location and quality of renal registries [24].

A systematic review of electronic literature databases was performed to identify the relevant data sources. It is possible that our analysis may have missed some data sources, although we did perform online searches of congresses and other websites, as well as conducting searches for proprietary data sources to identify any further sources that may not have been included in peer-review articles. Our searches were performed up until July 2016, so any data sources published after this date are not included in this analysis. At the time of the searches, the American Academy of Ophthalmology Intelligent Research in Sight (IRIS) registry had limited publications; as a result, this data source has not been highlighted in this study.

The bespoke grading system for data sources illustrates the breadth and type of data collected within each outcome of interest, but our study did not assess the data sources in terms of how the data are collected, the quality-check mechanisms in place, or how closely the data items collected compare with those recommended by the ICHOM guidelines. The assessment of the data sources may have been influenced by the amount of data available for a particular data source; for example, a source may have been graded 'poor' or 'unknown' for a particular outcome if retinal disease-related data from the source remains unpublished. For several of the data sources, we contacted the administrators to obtain further information that may not have been in the public domain to enable us to provide the most comprehensive assessment possible of these data sources. However, to limit the scope of the project, contacts were only pursued for a subset of the data sources. In addition, the completeness assessment was only undertaken for the multinational/international data sources, or sources from a shortlist of countries of interest. Therefore, the findings of this study should be interpreted with caution, as they are mostly relevant to international data sources or those from countries with published data. As a follow-up to this analysis, all the data sources identified in this SLR, including the proprietary data sources, could be assessed for their completeness.

## Conclusions

This study provides a comprehensive list of worldwide RWD sources for patients with retinal diseases treated with anti-VEGF therapies. Here, we show that there are many sources providing comprehensive data that can be used to conduct real-world studies. Although the sources vary in types of data items collected and the methods of data collection used, many of the RWD sources collect patient-relevant outcomes that provide important evidence in the field of ophthalmology.

## Method of literature search

RWD sources for patients with retinal diseases treated with anti-VEGF therapies were identified through an SLR. The SLR focused on searches using the electronic literature databases Embase (Elsevier) and MEDLINE (Ovid platform [Wolters-Kluwer], including MEDLINE In-Process & Other Non-Indexed Citations); the aim was to identify relevant articles describing RWD sources for retinal diseases, such as patient registries, hospital and insurance claims databases, electronic medical records, longitudinal and observational studies, as well as population-based surveys. The SLR was conducted in 2 stages: in the first stage, data sources (including multinational and international sources) outside of the USA were identified by applying a restriction to the search terms. The electronic search for the first stage was carried out in July 2016. In the second stage, a follow-up search was carried out in March 2017 specifically to identify articles describing retinal disease-related data sources within the USA.

In addition to searching the electronic literature databases, the following congress websites were used to identify relevant abstracts and posters published between 2012 and 2016: The International Society for Pharmacoeconomic Outcomes Research (ISPOR) European congress, Association of Research in Vision and Ophthalmology, World Ophthalmology Congress, European Society of Retina Specialists, Asia Pacific Vitreo-retina Society, and American Academy of Ophthalmology. Additional searches to identify country-specific RWD sources were conducted of clinical trial websites (clinicaltrials.gov and eudract.ema.europa.eu) and the World Health Organization registry list, as well as using the Google and Google Scholar search engines. To limit the scope of the study, the supplementary searches focused on only a restricted number of countries (Australia, Brazil, Canada, China, Denmark, Finland, France, Germany, Iceland, Italy, Japan, Norway, Spain, Sweden and the UK).

To identify large, proprietary databases that may not be referenced in publications, a grey literature search was conducted of the ISPOR website for both a list of databases and a list of vendors who may provide access to RWD sources. A Google search of RWD terms (such as ‘real-world data’, ‘real-world evidence’, ‘RWE’, etc.) was also undertaken.

## Additional file

Additional file 1: **Table S1.** Data sources and accessibility. (DOCX 32 kb)

## Data Availability

Not applicable.

## Abbreviations

BCVA: Best-corrected visual acuity
CNV: Choroidal neovascularization
DME: Diabetic macular edema
ICHOM: International Consortium for Health Outcomes Measurement
ISPOR: International Society for Pharmacoeconomic Outcomes Research
PRISMA: Preferred Reporting Items for Systematic Reviews and Meta-Analyses
RCT: Randomized controlled trial
RVO: Retinal vein occlusion
RWD: Real-world data
RWE: Real-world evidence
SLR: Systematic literature review
VEGF: Vascular endothelial growth factor
wAMD: Wet age-related macular degeneration
