# Supplementary material for: Real-world data in retinal diseases treated with anti-vascular endothelial growth factor (anti-VEGF) therapy – a systematic approach to identify and characterize data sources
Source: BMC Ophthalmol. 2019 Oct 16;19:206. doi: 10.1186/s12886-019-1208-9 (PMC6796465; doi:10.1186/s12886-019-1208-9)
Supplement: Supplementary file 1 — Table S1. Data sources and accessibility. (DOCX 32 kb) [file 12886_2019_1208_MOESM1_ESM.docx]

Additional file 1: Table S1 – Data sources and accessibility

| **Full name** | **Type of data source** | **Website** | **Accessibility to data*** | **Is the database linked to other data sources? (Y/N)** | ***Link to Medical records*** | **No. of patients enrolled** |
| --- | --- | --- | --- | --- | --- | --- |
| Melbourne Collaborative Cohort Study | Prospective cohort study | http://www.cancervic.org.au/research/epidemiology/health_2020 | Good | Unclear | - | 41501 |
| Hatoyama Cohort Study | Prospective cohort study | http://www.tmig.or.jp/eresearch/l02.html | Limited | Unclear | - | 742 |
| Blue Mountains Eye Study | Prospective cohort study | https://visionimpactinstitute.org/research/blue-mountains-eye-study/ http://www.cvr.org.au/ | Limited | Unclear | - | 3564 |
| Tromso Eye Study | Population-based longitudinal study | https://en.uit.no/prosjekter/prosjekt?p_document_id=80172 | Good | Unclear | - | 40051 |
| Four referral centres in Denmark | Medical records | N/A | Limited | Yes | Yes | N/A |
| The LUMINOUS programme | Prospective cohort study | http://www.novartisalconretina.com/docs/LUMINOUS_factsheet_Novartis.pdf | Limited | Yes | - | 4444 |
| Gloucestershire NHS ophthalmology department | Medical records | http://www.gloshospitals.nhs.uk/en/Wards-and-Departments/Departments/Ophthalmology/Research-Ophthalmology/Gloucestershire-Diabetic-Retinopathy-Research-Group/ <http://drscreening.org/pages/default.asp?id=16&sID=7> | Good | Yes | Yes | Unknown |
| UK Age-Related Macular Degeneration EMR Users Group | Retrospective cohort study | N/A | limited | Yes | Yes | - |
| Antioxydants, LIpids Essentiels, Nutrition et maladies OculaiRes | Prospective cohort study | N/A | limited | Yes | - | Unknown |
| European Genetic Database | Disease registry (active) | http://www.eugenda.org/ | Good | Yes | Yes | Unknown |
| Landschaftsverband Rheinland database | Disease registry (active) | http://www.lvr.de/de/nav_main/ | Limited | Unclear | - | 3328 |
| Age, Gene/Environment Susceptibility-Reykjavik Study | Prospective cohort study | http://www.hjartarannsokn.is/index.aspx?GroupId=346 | Good | Yes | - | 5764 |
| Fundus Autofluorescence in Age-related Macular Degeneration Study | Prospective cohort study | N/A | Limited | Unclear | - | - |
| Fight Retinal Blindness | Disease registry (active) | http://www.savesightinstitute.org.au/research/research-units/macula-research-group/#FRB http://sydney.edu.au/medicine/eye/research/macular/frb.php https://www.youtube.com/watch?v=ROyaDyIGvpE&feature=youtu.be | Good | Yes | Yes | - |
| Macular Disease Foundation | Disease registry (active) | http://www.mdfoundation.com.au/default.aspx | Limited | Unclear | - | 3170 |
| The National Ophthalmology Database | Disease registry (active) | https://www.nodaudit.org.uk/ | Good | Yes | Yes | - |
| The Comparison of Applied ophthalmological Tests for suspected AMD patIents (CAPTAIN) study | Retrospective cohort study | N/A | Limited | Yes | Yes | Unknown |
| Questionnaire study in ‘Association DMLA’ or ‘Retina France’ | Cross-sectional study | N/A | Limited | Unclear | - | Unknown |
| Beijing Eye Study | Population-based longitudinal study | N/A | Limited | No | - | 4439 |
| Australian Heart Eye Study | Prospective cohort study | N/A | Limited | Yes | Yes | 1680 |
| Study to Assess the Effectiveness of Existing Anti vascular Endothelial Growth Factor (Anti VEGF) in Patients With Wet Age-related Macular Degeneration | Retrospective cohort study | https://clinicaltrials.gov/ct2/show/NCT01447043 | Limited | Yes | Yes | - |
| A 2-Year, Phase IV, Multicentre, Observational Study of Ranibizumab 0.5mg in Patients with Neovascular Age-Related Macular Degeneration in Routine Clinical Practice | Prospective cohort study | N/A | limited | Unclear | - | - |
| Southwestern Ontario Database | Disease registry (passive) | N/A | Limited | Yes | - | >170,000 |
| English National Hospital Episode Statistics | Medical records | http://digital.nhs.uk/hes | Good | Yes | Yes | 245912 |
| British Ophthalmological Surveillance Unit | Medical records | https://www.rcophth.ac.uk/standards-publications-research/the-british-ophthalmological-surveillance-unit-bosu/ | Good | Yes | Yes | - |
| Medical Retina Clinic at King's College London | Medical records | N/A | Limited |  |  | Unknown |
| Creteil Intercommunal University Hospital Eye Clinic | Prospective cohort study | http://www.creteilophtalmo.fr/en/ | Good | Yes | Yes | - |
| Grampian University Hospitals | Medical records | N/A | Limited | Yes | Yes | Unknown |
| British Columbia (BC) Ministry of Health Databases | Administrative/Claims data | http://www2.gov.bc.ca/gov/content/health/conducting-health-research-evaluation/data-access-health-data-central | Good | Yes | Yes | 4.8 million |
| Gutenberg Health Study | Prospective cohort study | http://www.gutenberghealthstudy.org/ghs/overview.html | Good | Unclear | - | 15010 |
| Hisayama study | Prospective cohort study | N/A | Limited | Unclear | - | Unknown |
| Japan Medical Data Center | Administrative/Claims data | https://www.jmdc.co.jp/en/about/database.html | Good | Yes | Yes | 9851083 |
| Quebec prescription and medical claims databases | Administrative/Claims data | http://www.ramq.gouv.qc.ca/en/data-statistics/Pages/data-statistics.aspx | Limited | Yes | Yes | 800000 (regional population size) |
| South-east Scotland intravitreal ranibizumab treatment register (unnamed) | Disease registry (active) | N/A | Unclear | Yes | Yes | Unknown |
| The WHO Database of Adverse Drug Reactions | Disease registry (passive) | http://www.who-umc.org/DynPage.aspx?id=98082&mn1=7347&mn2=7252&mn3=7322&mn4=7326 | Good | Yes | Yes |  |
| Observation of Treatment Patterns With Lucentis in Approved Indications | Prospective cohort study | https://clinicaltrials.gov/ct2/show/NCT02194803 | Limited | Yes | - |  |
| The ILUVIEN Registry Safety Study | Disease registry (active) | https://clinicaltrials.gov/ct2/show/NCT01998412 | Good | Unclear | - |  |
| Bonn Opthalmology online network | Disease registry (active) | N/A | Unclear | Unclear | - |  |
| Swedish Macula Register | Disease registry (active) | N/A | Unclear | Unclear | - | 15606 |
| Medisoft | Medical records | http://www.medisoft.co.uk/ | Good | Yes | Yes | 13774 |
| Unnamed electronic database accessing the Belfast Health and Social Care Trust | Medical records | N/A | Unclear | Yes | Yes | Unknown |
| Swedish Lucentis Quality Registry | Disease registry (active) | N/A | Limited | Unclear | - | 475 |
| Medical Retina Service, St Thomas’ Hospital | Medical records | N/A | Unclear | Yes | Yes |  |
| Unnamed tertiary referral unit in the Midlands | Disease registry (active) | N/A | Limited | Yes | Yes |  |
| Unnamed tertiary referral clinic | Medical records | N/A | Unclear | Yes | Yes | - |
| * Good - Website describing data source/indications of accessibility or availability for research purposes, including annual reports;  Limited - Publication(s) suggests useful information available on data source but little further information available on how to access the data;  Unclear - Basic or minimal information available in published research on how to access the data | | | | | | |

| **Full name** | | **Type of data source** | **Website** | **Accessibility to data*** | **Is the database linked to other data sources? (Y/N)** | ***Link to Medical records*** | | **No. of patients enrolled** |
| --- | --- | --- | --- | --- | --- | --- | --- | --- |
|  |  |  |  |  |  |  |  |  |
| Age-Related Eye Disease Study | | Observational study (prospective) | https://nei.nih.gov/amd/background | Limited | Y | - | | 4757 |
| Behavioral Risk Factor Surveillance System | | Questionnaire/survey | <https://www.cdc.gov/brfss/> | Unclear | N | - | | - |
| Beaver Dam Eye Study | | Observational study (prospective) | <http://www.bdeyestudy.org/> | Limited | N | - | | 4926 |
| Bascom Palmer Eye Institute | | Disease registry (active) | [http://bascompalmer.org](http://bascompalmer.org/) | Good | N | - | | - |
| Carotenoids in Age-Related Eye Disease Study | | Observational study (prospective) | <https://www.nhlbi.nih.gov/whi/> | Limited | Y | - | | 1787 |
| Doheny Eye Institute | | Disease registry (active) | <http://www.doheny.org/> | Good | N | - | | - |
| Duke University Eye Center | | Disease registry (active) | [http://dukeeyecenter.duke.edu/research](https://www.dukehealth.org/treatments/eye-care/low-vision-rehabilitation) | Good | N | - | | - |
| i3 InVision Data Mart | | Electronic medical records | <http://ibi.uky.edu/i3-invision-data-mart> | Unclear | Y | Y | | > 15 million annual lives |
| IMS Health Real-World Data Medical Claims database | | Administrative/claims data | <http://csdmruk.cegedim.com/our-data/accessing-the-data.shtml> | Good | Y | Y | | Around 1 billion professional fee claims per year |
| Los Angeles Latino Eye Study | | Observational study (prospective) | <https://nei.nih.gov/latinoeyestudy/description> | Limited | Y | - | | 6881 |
| Multiethnic Study of Atherosclerosis | | Observational study (prospective) | <https://www.mesa-nhlbi.org/> | Good | Y | - | | 6814 |
| National Health and Nutrition Examination Survey | | Questionnaire/survey | <https://www.cdc.gov/nchs/nhanes/> | Good | Y | - | | 14464 |
| New England Eye Center | | Observational study (retrospective) | http://www.neec.com/ | Limited | N | - | | - |
| Nurses Health study | | Observational study (prospective) | <http://www.nurseshealthstudy.org/about-nhs/history> | Good | Y | - | > 280,000 | |
| Shiley Eye Center at UCSD | Observational study (prospective) | | [http://eyesite.ucsd.edu/research](http://eyesite.ucsd.edu/) | Limited | Y | Y | | - |
| Study of Osteoporotic Fractures - Incidence of AMD study | Observational study (prospective) | | <http://sof.ucsf.edu/interface/> | Good | Y | - | | 9704 |
| Vitreous Retina Macula Consultants of New York | Electronic medical records | | <https://vrmny.com/> | Good | Y | Y | | - |
| Wills Eye Hospital | | Electronic medical records | <https://www.willseye.org/> | Good | N | - | | > 250,000 annually |
| Wilmer Eye Institute | | Electronic medical records | <http://www.hopkinsmedicine.org/wilmer/> | Good | N | - | | - |
| * Good - Website describing data source/indications of accessibility or availability for research purposes, including annual reports; Limited - Publication(s) suggests useful information available on data source but little further information available on how to access the data; Unclear - Basic or minimal information available in published research on how to access the data | | | | | | | | |
